# Anatomical Variations of the Mandibular Buccal Shelf According to Sex, Facial Biotype, and Root Reference: A CBCT Study

**DOI:** 10.4317/jced.63384

**Published:** 2025-12-30

**Authors:** Estefania Patricia Flores-Villamarín, Mauricio Aguirre-Balseca, Marjory Elizabeth Vaca-Zapata, Karina Maria Salvatore Freitas, Eduardo Miranda

**Affiliations:** 1 D.D.S. Student. Department of Orthodontics, University of the Hemispheres, Quito, Ecuador. ORCID: 0009-0003-6598-2586; 2 D.D.S., M.Sc., Ph.D. Professor, Department of Endodontics, University of the Hemispheres, Quito, Ecuador. ORCID: 0000-0001-6597-9844; 3 D.D.S., M.Sc., Ph.D. Professor, Department of Orthodontics, University of the Hemispheres, Quito, Ecuador. ORCID: 0009-0009-5317-5131; 4 D.D.S., M.Sc., Ph.D. Professor, Department of Orthodontics, Ingá University Center Uningá, Maringá, Paraná, Brazil. ORCID: 0000-0001-9145-6334; 5 D.D.S. Professor. Department of Orthodontics, University of the Hemispheres, Quito, Ecuador. ORCID: 0009-0006-9441-6139

## Abstract

**Background:**

To evaluate, through cone-beam computed tomography (CBCT), the anatomical characteristics of the mandibular buccal shelf according to sex, facial biotype, hemiarch, and reference root, in order to determine bone availability for orthodontic mini-implant placement.

**Material and Methods:**

A cross-sectional observational study was conducted on 60 CBCT scans (480 roots) of patients aged 12-53 years. Measurements of angulation, horizontal bone thickness (4 mm and 6 mm), and vertical bone height (6 mm and 11 mm) were obtained at the mesial and distal roots of the mandibular first and second molars. Statistical analyses included t-test and one-way ANOVA (p &lt; 0.05).

**Results:**

The distal root of the mandibular second molar exhibited the highest bone dimensions (angulation 37°, horizontal thickness 17.3 mm at 4 mm and 10.0 mm at 6 mm, vertical height 5.4 mm at 6 mm and 6.7 mm at 11 mm), establishing it as the most favorable insertion site. In contrast, the mesial root of the first molar showed the lowest values. Sex-related analysis revealed slightly greater dimensions in males, with significant differences in horizontal thickness at 4 mm and vertical height at 11 mm (p = 0.05). No statistically significant differences were observed among facial biotypes or between hemiarches, although brachyfacial patients consistently showed the highest averages.

**Conclusions:**

The distal root of the mandibular second molar represents the most reliable anatomical site for mini-implant placement in the buccal shelf, regardless of sex or facial growth pattern. CBCT assessment remains essential for individualized planning and to minimize complications.

## Introduction

In recent years, orthodontics has incorporated new materials and techniques that have optimized treatment mechanics, among which mini-implants stand out as widely used temporary anchorage devices (1 , 2). These devices allow for more precise force control and, in many cases, reduce treatment time by enabling correction of vertical, sagittal, and transverse discrepancies (3). Since their introduction in clinical practice, primary stability has been recognized as a critical factor for success, depending on the mechanical interaction between the mini-implant surface and the surrounding bone (4). The mandibular buccal shelf has emerged as one of the most reliable areas for extra-alveolar mini-implant placement due to its cortical thickness and location in the posterior mandible, between the buccal roots of the molars and anterior to the external oblique ridge (1 , 3). Nevertheless, this region shows significant anatomical variability in both cortical thickness and vertical bone height, which raises debate regarding the most suitable insertion site (2 , 4). These anatomical differences are closely related to facial growth patterns, which directly influence bone morphology and therefore the stability of anchorage (5). Several studies have investigated the relationship between growth pattern and mandibular shelf characteristics using cone-beam computed tomography (CBCT). Aleluia et al. (6) demonstrated that both sex and skeletal pattern influence bone availability for mini-implant placement in this area. Similarly, Gandhi et al. (7) reported significant differences in mandibular shelf width and height associated with vertical and sagittal facial types. More recently, Eto et al. (8) evaluated buccal shelf thickness, bone height, and mandibular canal position, concluding that hyperdivergent patients had less bone availability compared to hypodivergent ones. Campoy et al. (9) also confirmed that mandibular shelf anatomy is strongly correlated with craniofacial morphology, emphasizing the need for individualized planning. In Latin American populations, Escobar et al. (1) analyzed mandibular shelf features in Colombian patients and reported significant differences according to sex and age, highlighting the importance of CBCT-based evaluation prior to insertion. Other investigations have reinforced the variability of mandibular shelf anatomy and the usefulness of CBCT for safe planning (10 - 12). Collectively, these studies indicate that mandibular bone morphology is not uniform and must be considered in a patient-specific context. CBCT has become the diagnostic tool of choice in this field, as it provides high-resolution three-dimensional images that allow for precise evaluation of bone availability, mandibular canal trajectory, and relationship with surrounding anatomical structures (13 - 15). Its clinical application has facilitated the identification of optimal sites for mini-implant placement, improved treatment predictability, and reduced the risk of complications. Despite the growing body of evidence, gaps remain regarding the characterization of the mandibular buccal shelf according to vertical growth patterns, particularly in Latin American populations. Addressing this issue is crucial to provide clinically relevant data that may guide orthodontic practice and optimize treatment planning. Therefore, the aim of this study is to evaluate, through cone-beam computed tomography, the anatomical characteristics of the mandibular buccal shelf in patients with different vertical growth patterns, in order to determine bone availability for safe orthodontic mini-implant placement.

## Material and Methods

**Figure 1:**
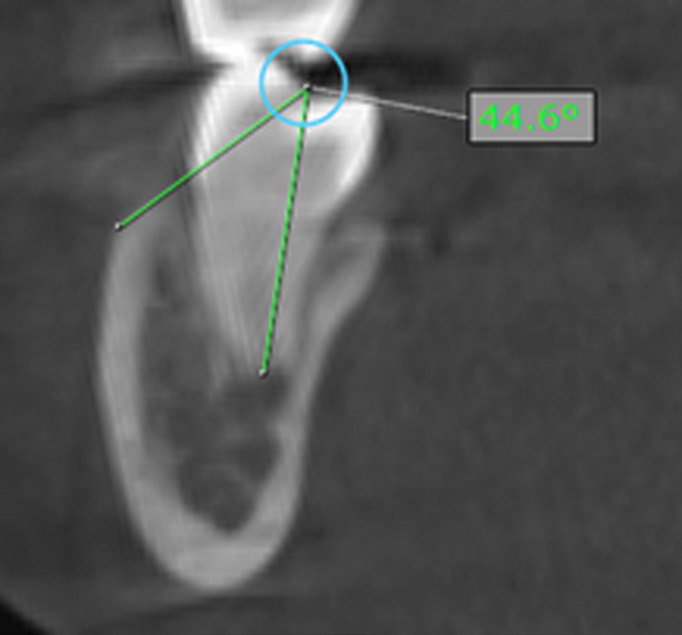
Measurement of the mandibular buccal shelf angulation.

This study was approved by the Ethics Committee of University of the Hemispheres, Quito, Ecuador (protocol n. CEUHE25-57) and all patients signed informed consent. This study was designed as an observational, descriptive, cross-sectional investigation. The sample consisted of cone-beam computed tomography (CBCT) scans obtained from the tomographic database of the University of the Hemispheres, Quito, Ecuador. The dataset included full-head CBCTs taken between 2022 and 2025. All scans were acquired with a Cone Beam Planmeca Promax MID Romexis Viewer 4.6.2.R 18/10/17, series TFMP 10360, with the following specifications: image size 200 × 17.6 cm, voxel size 400 µm, 120 kV, 6 mA. Inclusion criteria CBCT scans of the full skull Scans performed between 2022 and 2025 Patients with complete dentition up to the second molar Male and female patients Patients aged 12-53 years Exclusion criteria Pathologies (cysts, tumors, endodontically treated teeth, root resorption) Crowns on molars Severe bone resorption Facial trauma Previous orthognathic surgery Ongoing orthodontic or orthopedic treatment Syndromic patients From the initial database of 180 CBCTs, scans were classified according to the inclusion and exclusion criteria. A non-probabilistic convenience sample was established, resulting in 60 CBCTs that met all requirements. A total of 480 roots were measured, obtaining a homogeneous distribution among hypodivergent, normodivergent, and hyperdivergent patients. All scans were stored on an external hard drive. To determine the growth pattern, NemoStudio software was used. A lateral cephalogram was generated from the sagittal slice of each CBCT, and measurements were carried out following the cephalometric norms described by Ricketts, Steiner, and Jarabak. For the visualization of the mandibular buccal shelf, a transverse slice aligned to the long axis of the corresponding dental root was used. The CBCTs had a voxel resolution of 127 µm and slice thickness of 0.12 mm. Four reference sites were defined for measurement: Mesial root of the first molar Distal root of the first molar Mesial root of the second molar Distal root of the second molar Following the methodology proposed by Escobar-Correa et al. (1), the following parameters were assessed: 1. Angulation (Fig. 1): the angle between the long axis of the molar and a tangent to the most external surface of the mandibular buccal shelf (1 , 4).

1

**Figure 2:**
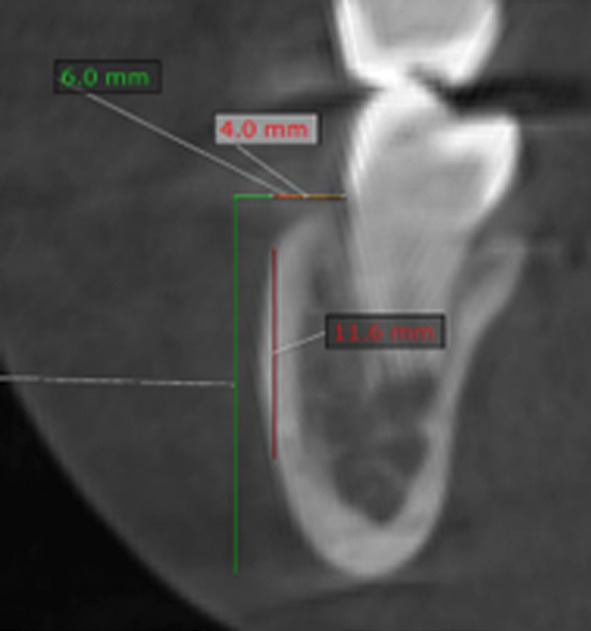
Measurement of mandibular buccal shelf height.

2. Height (Fig. 2): measured on cortical and medullary bone using two horizontal reference lines located 4 mm and 6 mm apical to the cementoenamel junction, intersecting with two vertical lines extended to the outermost limit of the mandibular cortical plate (1).

2

**Figure 3:**
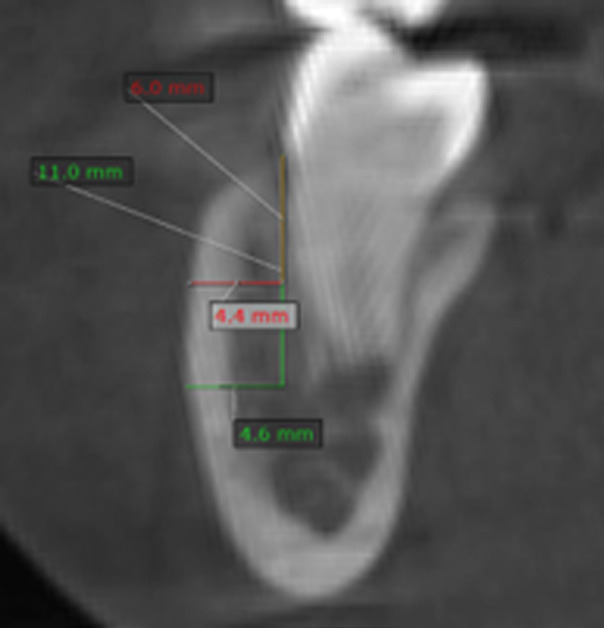
Measurement of mandibular buccal shelf thickness.

3. Thickness (Fig. 3): measured by tracing two vertical lines at 6 mm and 11 mm from the cemento-enamel junction, parallel to the X-axis, and two horizontal lines to the external border of the cortical bone (1).

3

All data were recorded in Microsoft Excel. Measurements were assigned to each facial biotype and classified according to the first and second molars, left and right sides. To ensure reliability, two sets of measurements were taken. Ten randomly selected CBCTs were re-measured after an 8-day interval by an experienced radiologist. This intra-examiner reliability procedure was used to enhance methodological validity and consistency. After data collection, results were compiled in Microsoft Excel 2019 and exported to SPSS version 23 (IBM®, Spanish version) for statistical analysis. Descriptive statistics (means, standard deviations, and measures of dispersion) were calculated. The level of significance was set at p &lt; 0.05. The Student's t-test was applied for comparisons between two groups, and one-way ANOVA was used for comparisons among more than two groups.

## Results

Overall, the measurements of the mandibular buccal shelf showed comparable values across groups, with dispersions within the expected range. The only statistically significant difference was found in the vertical measurement at 11 mm in relation to sex, with males presenting higher values (p = 0.03). No statistically significant differences were observed among facial biotypes for any of the evaluated variables. Right Second Molar (Tables 1,2) For the distal root, mean values were homogeneous across groups, with a significant sex-related difference only in the vertical measurement at 11 mm (greater in males).

Table 1

Table 2

The brachyfacial pattern showed the highest mean values in most variables, although without statistical significance. For the mesial root, no significant differences were found by sex or facial biotype. Males tended to present higher mean values, especially at 11 mm vertical (p = 0.07), close to the threshold of significance. Right First Molar (Tables 3,4) At the distal root, no significant differences were found between sexes or facial biotypes.

Table 3

Males consistently showed higher mean values, although not significant. The brachyfacial group recorded the highest means for angular and horizontal measures, while the dolichofacial group showed higher vertical values. At the mesial root, no significant differences were found, although males presented slightly higher averages. Global mean values were: angle 18°, horizontal 4 mm 2.4 mm, horizontal 6 mm 0.7 mm, vertical 6 mm 0.9 mm, and vertical 11 mm 1.7 mm. Dispersion was greater for horizontal and vertical measures. Left Second Molar (Tables 5,6) For the distal root, results were similar between sexes and biotypes, except for a marginal difference at 11 mm vertical (p = 0.050), favoring males.

Table 4

Table 5

Table 6

For the mesial root, no significant differences were observed, although males again showed higher means. The brachyfacial group presented the highest vertical values at 6 mm (p = 0.030), followed by mesofacial, while dolichofacial showed the lowest. Left First Molar (Tables 7,8) At the distal root, no significant sex-related differences were detected, although the brachyfacial group consistently showed higher averages.

Table 7

Table 8

Global means were: angle 21°, horizontal 4 mm 7.2 mm, horizontal 6 mm 1.6 mm, vertical 6 mm 1.8 mm, and vertical 11 mm 3.4 mm. At the mesial root, no significant differences were observed by sex or biotype. The brachyfacial group once again presented the highest averages, suggesting a more prominent buccal shelf in this pattern. Comparative Analysis When analyzing global data (Table 9), males consistently showed higher mean values across all dimensions, although only the horizontal distance at 4 mm and the vertical distance at 11 mm approached statistical significance (p = 0.05).

Table 9

These findings suggest a tendency for males to present a more prominent buccal shelf. No significant differences were observed among facial biotypes (Table 10).

Table 10

However, brachyfacial patients consistently showed the highest means, followed by mesofacial, while dolichofacial subjects presented the lowest values, consistent with their thinner mandibular morphology. Comparison by hemiarch (Table 11) showed no significant asymmetries between right and left sides, although the left hemiarch tended to present slightly higher horizontal values.

Table 11

Finally, analysis by reference root (Table 12) revealed statistically significant differences for all variables (p &lt; 0.01). The buccal shelf was most prominent at the distal root of the second molar, whereas the mesial root of the first molar presented the lowest values.

Table 12

This distribution highlights the distal area of the mandibular second molar as the most favorable site for mini-implant placement. Table 13 summarizes the mean values and confidence intervals for each variable, providing clinically relevant reference ranges for treatment planning.

Table 13

## Discussion

The present study evaluated the anatomical characteristics of the mandibular buccal shelf according to sex, facial biotype, and reference root using cone-beam computed tomography (CBCT). Overall, the results demonstrated consistent patterns of bone availability, with statistically significant differences primarily associated with the reference root and, to a lesser extent, with sex. No significant differences were observed among facial growth patterns or hemiarches. These findings provide clinically relevant insights for planning mini-implant insertion in the mandibular buccal shelf region. Influence of the reference root The most notable finding of this investigation was that the distal root of the mandibular second molar exhibited the highest values for angulation (37°), horizontal bone thickness (17.3 mm at 4 mm and 10.0 mm at 6 mm), and vertical bone height (5.4 mm at 6 mm and 6.7 mm at 11 mm). In contrast, the mesial root of the first molar presented the lowest dimensions across all parameters. This distribution highlights the distal root of the second molar as the most favorable site for mini-implant placement. These results are consistent with García-Gonzales &amp; Ruiz (4), who also identified significantly greater bone depth and thickness in the distal root of the second molar compared with mesial roots, confirming the biomechanical advantages of this site. Similarly, Nucera et al. (12) and Hong et al. (16) reported that the distal aspect of the mandibular second molar provides sufficient cortical support to ensure primary stability and minimize the risk of root interference. Sex-related differences Although global averages were higher in males than in females, statistically significant differences were limited to horizontal bone thickness at 4 mm and vertical height at 11 mm (p = 0.05). Males consistently presented greater values, suggesting a trend toward a more prominent buccal shelf. Eto et al. (8) also found higher vertical bone height in male patients, while reporting no significant sex differences in cortical thickness. These findings are in line with Shrivastava et al. (17), who demonstrated that males exhibited greater bone height in the distal roots of mandibular molars. Clinically, these results imply that sex-related variability should be considered during mini-implant planning, particularly in borderline cases with limited bone availability. Facial growth pattern No statistically significant differences were observed among brachyfacial, mesofacial, and dolichofacial patients across all evaluated parameters. Nonetheless, brachyfacial patients consistently exhibited the highest mean values, followed by mesofacial and dolichofacial groups. This tendency is in agreement with Matias et al. (11) and Ramasamy et al. (18), who noted increased cortical thickness and bone availability in brachyfacial individuals, despite the absence of strong statistical associations. From a clinical perspective, this suggests that while facial growth pattern may not be a decisive predictor of mandibular buccal shelf dimensions, brachyfacial patients often present more favorable anatomical conditions for mini-implant insertion. Laterality (hemiarch comparison) The comparison between right and left hemiarches showed no significant asymmetries, although the left side tended to present slightly greater horizontal values. These findings are consistent with Escobar-Correa et al. (1) and Macrì &amp; Festa (19), who also reported symmetrical mandibular buccal shelf morphology between sides, reinforcing the notion that clinicians can safely plan mini-implant insertion on either hemiarch without concern for significant anatomical differences. Clinical implications The identification of the distal root of the mandibular second molar as the most favorable site for mini-implant insertion has important clinical implications. This location offers sufficient cortical thickness and vertical bone height to ensure primary stability, even under complex mechanics such as molar distalization, anterior retraction, or vertical control. Furthermore, the absence of significant associations with facial biotypes suggests that this insertion site may be universally reliable across different craniofacial morphologies. However, clinicians should remain cautious in female patients and dolichofacial individuals, who may present slightly reduced bone availability, requiring individualized assessment through CBCT. Strengths and limitations A major strength of this study lies in the use of CBCT, which provides three-dimensional, high-resolution evaluation of the mandibular buccal shelf and allows precise measurement of angulation, height, and thickness. Additionally, by including patients across different growth patterns and sexes, the study provides a comprehensive overview of anatomical variability. Nevertheless, limitations include the convenience sampling method and lack of stratification by ethnicity, which may restrict generalizability. Future studies with larger and more diverse populations are recommended to confirm these findings and to establish standardized clinical guidelines for mini-implant placement in the mandibular buccal shelf region.

## Conclusions

This CBCT study demonstrated that the distal root of the mandibular second molar provides the most favorable anatomical conditions for mini-implant placement, with greater angulation, bone thickness, and height compared with other sites. No significant differences were found between facial growth patterns or hemiarches, while males showed slightly higher values than females. Clinically, the distal aspect of the second molar can be considered the most reliable site for mandibular buccal shelf mini-implants, although individualized CBCT evaluation remains essential for safe and predictable outcomes.

## Figures and Tables

**Table 1:** Characteristics of the mandibular buccal shelf according to sex and facial biotype for the right second molar – distal.

	ANGLE		4mm HORIZONTAL		6mm HORIZONTAL		6mm VERTICAL		11mm VERTICAL	
Sex	Mean	SD	Mean	SD	Mean	SD	Mean	SD	Mean	SD
Females	37.0	10.6	16.2	6.4	8.1	8.0	5.3	1.4	6.3	1.5
Males	37.2	6.8	18.3	3.6	12.1	6.3	5.1	1.8	7.2	1.4
p	0.931		0.186		0.063		0.682		0.030*	
Mesofacial	38.2	8.4	17.3	4.6	10.0	7.7	5.2	1.7	6.7	1.4
Dolichofacial	34.5	10.6	15.4	7.2	8.1	7.7	5.1	1.3	6.3	1.8
Brachyfacial	38.4	9.2	17.8	4.9	10.0	7.8	5.4	1.7	6.6	1.5
Total	37.0	9.5	16.8	5.7	9.4	7.7	5.2	1.5	6.6	1.6
p	0.331		0.381		0.679		0.812		0.634	

* Statistically significant for p<0.05

**Table 2:** Characteristics of the mandibular buccal shelf according to sex and facial biotype for the right second molar – mesial.

	ANGLE		4mm HORIZONTAL		6mm HORIZONTAL		6mm VERTICAL		11mm VERTICAL	
Sex	Mean	SD	Mean	SD	Mean	SD	Mean	SD	Mean	SD
Females	31.7	8.6	12.8	8.7	5.8	7.0	5.3	1.4	5.4	1.6
Males	31.5	5.5	15.1	5.6	8.6	7.5	5.1	1.8	6.3	2.0
p	0.940		0.300		0.170		0.770		0.070	
Mesofacial	31.7	6.6	14.0	7.2	6.8	7.7	5.2	1.7	5.6	1.8
Dolichofacial	30.4	8.8	13.6	8.8	6.5	7.1	5.1	1.3	5.8	1.9
Brachyfacial	32.8	7.9	13.0	7.8	6.8	7.2	5.4	1.7	5.6	1.7
Total	31.6	7.7	13.5	7.9	6.7	7.2	5.2	1.5	5.7	1.8
p	0.641		0.918		0.993		0.872		0.970	

2

**Table 3:** Characteristics of the mandibular buccal shelf according to sex and facial biotype for the right first molar – distal.

	ANGLE		4mm HORIZONTAL		6mm HORIZONTAL		6mm VERTICAL		11mm VERTICAL	
Sex	Mean	SD	Mean	SD	Mean	SD	Mean	SD	Mean	SD
Females	21.0	7.0	6.0	7.6	1.3	3,7	1.6	1.4	2.9	1.9
Males	21.8	3.9	7.4	7.4	2.5	5,0	1.8	1.3	3.7	2.1
p	0.640		0.500		0.290		0.640		0.200	
Mesofacial	22.1	4.4	5.4	7.2	1.8	4,4	1.4	1.2	2.9	1.8
Dolichofacial	18.9	5.6	5.4	7.3	0.8	3,5	1.4	1.2	3.0	2.1
Brachyfacial	22.7	7.7	8.4	8.0	2.4	4,5	2.1	1.5	3.6	2.1
Total	21.2	6.2	6.4	7.5	1.7	4,1	1.7	1.4	3.2	2.0
p	0.117		0.346		0.468		0.170		0.429	

3

**Table 4:** Characteristics of the mandibular buccal shelf according to sex and facial biotype for the right first molar – mesial.

	ANGLE		4mm HORIZONTAL		6mm HORIZONTAL		6mm VERTICAL		11mm VERTICAL	
Sex	Mean	SD	Mean	SD	Mean	SD	Mean	SD	Mean	SD
Females	36.7	11.5	17.4	6.0	9.8	7.5	5.6	1.3	6.6	1.4
Males	37.2	7.5	18.9	3.9	12.7	7.0	5.6	1.9	7.4	1.5
p	0.870		0.310		0.160		0.980		0.050	
Mesofacial	37.8	8.9	18.7	3.2	11.5	7.2	5.4	1.7	7.2	1.4
Dolichofacial	34.3	12.1	16.1	7.5	8.7	7.4	5.3	1.2	6.5	1.6
Brachyfacial	38.6	9.7	18.7	4.6	11.8	7.6	6.0	1.5	6.9	1.5
Total	36.9	10.3	17.9	5.5	10.7	7.4	5.6	1.5	6.9	1.5
p	0.378		0.225		0.350		0.230		0.409	

4

**Table 5:** Characteristics of the mandibular buccal shelf according to sex and facial biotype for the left second molar – distal.

	ANGLE		4mm HORIZONTAL		6mm HORIZONTAL		6mm VERTICAL		11mm VERTICAL	
Sex	Mean	SD	Mean	SD	Mean	SD	Mean	SD	Mean	SD
Females	21.0	7.0	6.0	7.6	1.3	3,7	1.6	1.4	2.9	1.9
Males	21.8	3.9	7.4	7.4	2.5	5,0	1.8	1.3	3.7	2.1
p	0.640		0.500		0.290		0.640		0.200	
Mesofacial	22.1	4.4	5.4	7.2	1.8	4,4	1.4	1.2	2.9	1.8
Dolichofacial	18.9	5.6	5.4	7.3	0.8	3,5	1.4	1.2	3.0	2.1
Brachyfacial	22.7	7.7	8.4	8.0	2.4	4,5	2.1	1.5	3.6	2.1
Total	21.2	6.2	6.4	7.5	1.7	4,1	1.7	1.4	3.2	2.0
p	0.117		0.346		0.468		0.170		0.429	

5

**Table 6:** Characteristics of the mandibular buccal shelf according to sex and facial biotype for the left second molar – mesial.

	ANGLE		4mm HORIZONTAL		6mm HORIZONTAL		6mm VERTICAL		11mm VERTICAL	
Sex	Mean	SD	Mean	SD	Mean	SD	Mean	SD	Mean	SD
Females	31.7	8.8	14.6	6.9	6.5	6.7	4.0	1.7	5.8	1.3
Males	32.6	5.7	16.1	5.6	10.1	7.2	4.1	1.9	6.5	1.9
p	0.700		0.400		0.070		0.770		0.140	
Mesofacial	32.1	6.3	16.2	4.0	9.1	6.4	4.1	1.9	6.2	1.7
Dolichofacial	30.7	9.2	13.7	8.4	6.7	6.8	3.7	1.5	5.8	1.3
Brachyfacial	33.1	8.1	15.3	6.4	7.3	7.9	4.3	1.9	6.1	1.7
Total	32.0	7.9	15.1	6.5	7.7	7.0	4.0	1.7	6.1	1.5
p	0.633		0.478		0.538		0.548		0.659	

6

**Table 7:** Characteristics of the mandibular buccal shelf according to sex and facial biotype for the left first molar – distal.

	ANGLE		4mm HORIZONTAL		6mm HORIZONTAL		6mm VERTICAL		11mm VERTICAL	
Sex	Mean	SD	Mean	SD	Mean	SD	Mean	SD	Mean	SD
Females	21.0	6.8	7.4	7.7	1.2	3.8	1.8	1.4	3.4	1.7
Males	21.9	4.1	6.8	7.4	2.4	4.8	1.6	1.2	3.5	1.7
p	0.560		0.780		0.320		0.570		0.850	
Mesofacial	22.4	4.0	6.5	6.9	1.6	3.9	1.6	1.1	3.4	1.5
Dolichofacial	18.9	5.8	6.5	8.0	0.0	0.0	1.4	1.0	3.0	1.4
Brachyfacial	22.5	7.4	8.5	8.1	3.2	5.8	2.3	1.7	3.8	2.1
Total	21.3	6.0	7.2	7.6	1.6	4.2	1.8	1.3	3.4	1.7
p	0.096		0.643		0.030*		0.122		0.371	

* Statistically significant for p<0.05

**Table 8:** Characteristics of the mandibular buccal shelf according to sex and facial biotype for the left first molar – mesial.

	ANGLE		4mm HORIZONTAL		6mm HORIZONTAL		6mm VERTICAL		11mm VERTICAL	
Sex	Mean	SD	Mean	SD	Mean	SD	Mean	SD	Mean	SD
Females	21.0	6.8	7.4	7.7	1.2	3.8	1.8	1.4	3.4	1.7
Males	21.9	4.1	6.8	7.4	2.4	4.8	1.6	1.2	3.5	1.7
p	0.560		0.780		0.320		0.570		0.850	
Mesofacial	22.4	4.0	6.5	6.9	1.6	3.9	1.6	1.1	3.4	1.5
Dolichofacial	18.9	5.8	6.5	8.0	0.0	0.0	1.4	1.0	3.0	1.4
Brachyfacial	22.5	7.4	8.5	8.1	3.2	5.8	2.3	1.7	3.8	2.1
Total	21.3	6.0	7.2	7.6	1.6	4.2	1.8	1.3	3.4	1.7
p	0.096		0.643		0.030*		0.122		0.371	

* Statistically significant for p<0.05

**Table 9:** Characteristics of the mandibular buccal shelf according to sex.

SEX	ANGLE	4mm HORIZONTAL	6mm HORIZONTAL	6mm VERTICAL	11mm VERTICAL
Females	26.8	9.8	4.1	3.0	4.2
Males	27.7	11.2	6.4	3.1	4.9
p	0.610	0.050	0.070	0.670	0.050

9

**Table 10:** Characteristics of the mandibular buccal shelf according to facial biotype.

BIOTYPE	ANGLE	4mm HORIZONTAL	6mm HORIZONTAL	6mm VERTICAL	11mm VERTICAL
Mesofacial	28.0	10.5	5.3	2.9	4.5
Dolichofacial	25.1	9.4	4.0	2.8	4.1
Brachyfacial	28.1	10.8	5.3	3.3	4.6
p	0.270	0.300	0.120	0.400	0.380

10

**Table 11:** Characteristics of the mandibular buccal shelf according to hemiarch.

HEMIARCH	ANGLE	4mm HORIZONTAL	6mm HORIZONTAL	6mm VERTICAL	11mm VERTICAL
Right	27.0	9.8	4.6	3.0	4.3
Left	27.2	10.7	5.1	3.1	4.5
p	0.710	0.100	0.110	0.620	0.570

11

**Table 12:** Characteristics of the mandibular buccal shelf according to reference root.

REFERENCE ROOT	ANGLE	4mm HORIZONTAL	6mm HORIZONTAL	6mm VERTICAL	11mm VERTICAL
Distal second molar	37.0	17.3	10.0	5.4	6.7
Mesial second molar	31.8	14.3	7.2	4.0	5.9
Distal first molar	21.3	6.8	1.6	1.7	3.3
Mesial first molar	18.3	2.5	0.5	1.0	1.7
p	0.000*	0.000*	0.000*	0.000*	0.000*

* Statistically significant for p<0.05

**Table 13:** Summary with mean value and confidence interval for each variable.

47 DISTAL	ANGLE	37±2.4
	4mm HORIZONTAL	16.8±1.4
	6mm HORIZONTAL	9.4±1.9
	6mm VERTICAL	5.2±0.4
	11mm VERTICAL	6.6±0.4
47 MESIAL	ANGLE	31.6±2
	4mm HORIZONTAL	13.5±2
	6mm HORIZONTAL	6.7±1.8
	6mm VERTICAL	4±0.5
	11mm VERTICAL	5.7±0.4
46 DISTAL	ANGLE	21.2±1.6
	4mm HORIZONTAL	6.4±1.9
	6mm HORIZONTAL	1.7±1
	6mm VERTICAL	1.7±0.3
	11mm VERTICAL	3.2±0.5
46 MESIAL	ANGLE	18±1.4
	4mm HORIZONTAL	2.4±1.4
	6mm HORIZONTAL	0.7±0.7
	6mm VERTICAL	0.9±0.2
	11mm VERTICAL	1.7±0.5
37 DISTAL	ANGLE	36.9±2.6
	4mm HORIZONTAL	17.9±1.4
	6mm HORIZONTAL	10.7±1.9
	6mm VERTICAL	5.6±0.4
	11mm VERTICAL	6.9±0.4
37 MESIAL	ANGLE	32±2
	4mm HORIZONTAL	15.1±1.6
	6mm HORIZONTAL	7.7±1.8
	6mm VERTICAL	4±0.4
	11mm VERTICAL	6.1±0.4
36 DISTAL	ANGLE	21.3±1.5
	4mm HORIZONTAL	7.2±1.9
	6mm HORIZONTAL	1.6±1.1
	6mm VERTICAL	1.8±0.3
	11mm VERTICAL	3.4±0.4
36 MESIAL	ANGLE	18.6±1.5
	4mm HORIZONTAL	2.5±1.5
	6mm HORIZONTAL	0.4±0.5
	6mm VERTICAL	1±0.2
	11mm VERTICAL	1.7±0.4

13

## Data Availability

The datasets used and/or analyzed during the current study are available from the corresponding author.
